# The impact of hotspot-targeted interventions on malaria transmission: study protocol for a cluster-randomized controlled trial

**DOI:** 10.1186/1745-6215-14-36

**Published:** 2013-02-02

**Authors:** Teun Bousema, Jennifer Stevenson, Amrish Baidjoe, Gillian Stresman, Jamie T Griffin, Immo Kleinschmidt, Edmond J Remarque, John Vulule, Nabie Bayoh, Kayla Laserson, Meghna Desai, Robert Sauerwein, Chris Drakeley, Jonathan Cox

**Affiliations:** 1 Department of Immunology & Infection; Faculty of Infectious and Tropical Diseases, London School of Hygiene and Tropical Medicine, London, UK; 2 Radboud University Nijmegen Medical Centre, Nijmegen, the Netherlands; 3 Department of Disease Control; Faculty of Infectious and Tropical Diseases, London School of Hygiene and Tropical Medicine, London, UK; 4 MRC Centre for Outbreak Analysis & Modelling, Department of Infectious Disease Epidemiology, Imperial College London, London, UK; 5 MRC Tropical Epidemiology Group, Department of Infectious Disease Epidemiology, London School of Hygiene and Tropical Medicine, London, UK; 6 Department of Parasitology, Biomedical Primate Research Centre, Rijswijk, The Netherlands; 7 Kenya Medical Research Institute, Centre for Global Health Research, Kisumu, Kenya; 8 Centers for Disease Control and Prevention, Division of Parasitic Diseases and Malaria, Atlanta, GA, USA

**Keywords:** *Anopheles*, elimination, epidemiology, eradication, falciparum, heterogeneity, immunology, malaria, molecular, transmission

## Abstract

**Background:**

Malaria transmission is highly heterogeneous in most settings, resulting in the formation of recognizable malaria hotspots. Targeting these hotspots might represent a highly efficacious way of controlling or eliminating malaria if the hotspots fuel malaria transmission to the wider community.

**Methods/design:**

Hotspots of malaria will be determined based on spatial patterns in age-adjusted prevalence and density of antibodies against malaria antigens apical membrane antigen-1 and merozoite surface protein-1. The community effect of interventions targeted at these hotspots will be determined. The intervention will comprise larviciding, focal screening and treatment of the human population, distribution of long-lasting insecticide-treated nets and indoor residual spraying. The impact of the intervention will be determined inside and up to 500 m outside the targeted hotspots by PCR-based parasite prevalence in cross-sectional surveys, malaria morbidity by passive case detection in selected facilities and entomological monitoring of larval and adult *Anopheles* populations.

**Discussion:**

This study aims to provide direct evidence for a community effect of hotspot-targeted interventions. The trial is powered to detect large effects on malaria transmission in the context of ongoing malaria interventions. Follow-up studies will be needed to determine the effect of individual components of the interventions and the cost-effectiveness of a hotspot-targeted approach, where savings made by reducing the number of compounds that need to receive interventions should outweigh the costs of hotspot-detection.

**Trial registration:**

NCT01575613. The protocol was registered online on 20 March 2012; the first community was randomized on 26 March 2012.

## Background

The transmission of infectious agents is highly heterogeneous in space and time. For many infectious diseases, a small number of human hosts are most frequently or most heavily infected while the majority of a local population is much less affected [1-4]. In malaria, this heterogeneity of disease transmission often results in variation in malaria incidence within small areas [5-10]. In some settings the non-random distribution of malaria incidence between households appears to conform to the ‘20/80 rule’ [2], whereby approximately 20% of a host population contributes 80% of the cases of an infectious organism [5,9]. The factors underlying the micro-epidemiology of malaria are not fully understood but include variation in distance from the nearest mosquito breeding site [5-9,11], wind direction [12], house construction features [6,8,9,13,14], human behavioural [7,8,13] and genetic factors [7,8,15].

Heterogeneity in malaria transmission has implications for malaria control. Individuals who are bitten most often are most likely to be infected and can amplify transmission by infecting a large number of mosquitoes with malaria parasites. Estimates of the basic reproductive number (*R*_0_), a central concept in infectious disease epidemiology defined as the average number of secondary cases arising in a susceptible population as a result of a single human case over the course of their infection, are sensitive to assumptions of heterogeneous mosquito exposure. *R*_0_ may be four times higher when heterogeneous mosquito exposure, as opposed to homogeneous exposure, is considered [2,4,16].

The large influence of heterogeneous exposure on malaria transmission also suggests that interventions targeting areas of comparatively high exposure can be highly effective. Woolhouse and colleagues suggested that, depending on the costs of identifying hotspots of transmission, treating the core 20% might be preferable to non-targeted interventions on economic grounds [2]. If hotspots fuel transmission to a wider geographical region, community protection may be achieved by targeting those individuals that are most important for disease transmission. This hotspot-targeted approach will be most (cost) effective if the assumption that hotspots fuel transmission in surrounding areas is correct – and then only if such hotspots can be reliably detected [4]. Several approaches to identify hotspots of malaria transmission have been proposed in recent years. Annual incidence of clinical malaria is a frequently used indicator of hotspots of malaria transmission [8-10] but is affected by a differential acquisition of protective immune responses inside and outside hotspots [17,18]. Geographical clustering of asymptomatic parasite carriage may be a more stable indicator of hotspots of transmission [10] and in areas of moderate or low endemicity hotspots might be most readily detected using serological markers of malaria exposure [9,10,19-22]. In an area of moderate endemicity in Tanzania, serological data have been used to identify clinically and entomologically confirmed hotspots of malaria transmission with 96% sensitivity and 82% specificity [9].

This manuscript describes a methodological approach to identifying hotspots of malaria transmission and a protocol for the evaluation of a hotspot-targeted intervention. The aim of this intervention study is to determine whether the simultaneous roll-out of interventions in hotspots of malaria transmission has a community-wide effect that extends beyond the hotspot boundaries and results in local reduction and possibly elimination of malaria.

## Methods/design

### Study area

The study will be conducted in highland fringe localities (1400 m to 1600 m altitude) in Rachuonyo South District, Western Kenya (34.75 to 34.95°E, 0.41 to 0.52°S). The predominant ethnicity in Rachuonyo is Luo. Local residents depend upon farming, cattle and goat herding for subsistence [23]. Compounds comprise an average of two houses (25th to 75th percentile 1 to 3) and are distributed broadly across a rolling landscape intersected with small streams and rivers. The main malaria vectors in the area are *Anopheles gambiae s.s.*, *An. arabiensis*, and *An. funestus*. Malaria transmission is seasonal, with two peaks in malaria cases reflecting the bimodal rainfall pattern; a peak corresponding to the heaviest rainfall typically occurs between March and June and there is a smaller peak between October and November each year. Most malaria is caused by *Plasmodium falciparum*[23]. Community cross-sectional surveys conducted in 2010 indicated parasite prevalence averaging 14.8% in the general population but varying between localities from 0% and 51.5%. School surveys carried out in primary schools in the same year indicated an average parasite prevalence of 25.8% in 7 to 18 year olds (minimum and maximum for individual schools 0 to 71.4%). Insecticide-treated nets (ITNs) have been promoted by the Ministry of Public Health and Sanitation for many years and distribution campaigns have taken place through antenatal and child health clinics, reaching net ownership for under 5s of 82.7%, as determined in surveys in 2010 (unpublished data). In addition, community-wide mass distribution of ITNs was undertaken by the Division of Malaria Control (DOMC) in 2011. Indoor residual spraying (IRS) with a pyrethroid was first carried out in Rachuonyo South in mid 2008 with financial support of the US President’s Malaria Initiative. Reported house coverage with IRS in Rachuonyo South was estimated at 70.3% in 2009 and 74.3% in 2010.

### Sampling strategy to identify hotspots of transmission

We will select a 5 × 20 km (100 km^2^) area within which results from recent community and school malaria surveys suggest highly heterogeneous malaria exposure. The study area will be divided into 400 cells of 500 × 500 m that will be further subdivided into four sub-cells of 250 × 250 m.

All structures in the area have been geo-located using contemporaneous high-resolution satellite data (Quickbird; DigitalGlobe Services, Inc., Denver, CO, USA), which were acquired and processed using standard digital image processing techniques (ENVI 4.8, Exelis Visual Information Solutions, McLean, VA, USA). Pan-sharpened colour images were then imported into a geographic information system (ArcGIS 9.2; Environmental Systems Research Institute, Redlands, CA, USA) and all structures digitized manually, giving a total of 8,632 structures with a median of 45 (25th to 75th percentile, 35 to 52) per 500 × 500 m cell. We aim to obtain measurements from ≥50 individuals per 500 × 500 m cell, since estimates of sero-conversion rates from fewer than 50 observations from all age groups combined are likely to be unreliable [9]. To maximize the discriminative power of serological markers of exposure, we will sample individuals in predefined age strata (≤5 years; 6 to 10 years; 11 to 15 years; 16 to 25 years and >25 years). For logistical reasons, our unit of sampling will be the compound.

To limit the chances of two selected structures belonging to the same compound, an iterative sampling approach will be used that involves randomly selecting a ‘seed’ structure and then removing all closely neighbouring structures (within 50 m) from the sample universe before proceeding to select a second structure. This process will be repeated until all possible ‘non-neighbouring’ structures have been selected. From the resulting list of eligible structures a sample of 16 structures will be chosen from each 500 × 500 m cell. To ensure maximum geographical coverage, at least one compound will be selected from each 250 × 250 m sub-cell, while the number of compounds selected from each of the sub-cells will be weighted by the structure density in these sub-cells.

All other structures in which people sleep and which are associated with each selected compound will be included. The target number of 50 observations per 500 × 500 m cell is chosen irrespective of the population density of the cells.

### Data collection and measurements to identify hotspots of transmission

#### Enumeration

**Figure 1:**
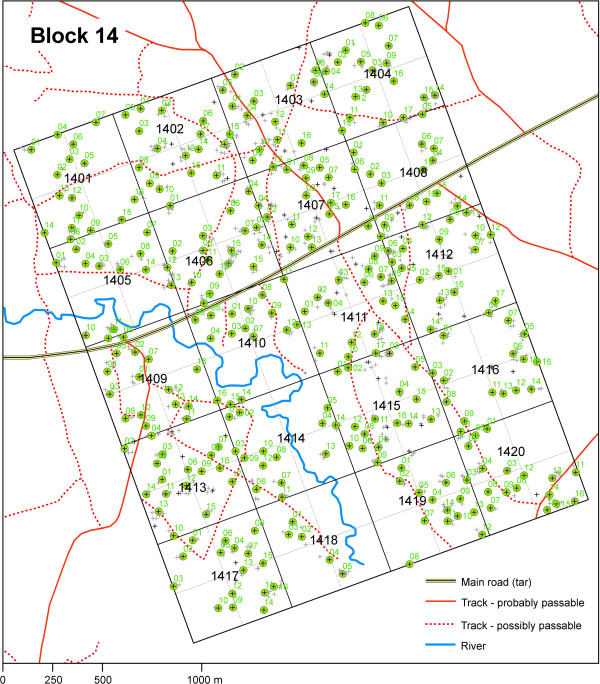
**Overview map of one block in the study area comprising 20 cells.** A map of a 2 × 2.5 km section of the study area that comprises 20 500 × 500 m cells and 80 sub-cells. Cell numbers are given in black bold letters; grey crosses indicate structures; green circles with black crosses indicate selected and numbered households. Rivers and roads are indicated in the map as given in the legend.

For planning purposes, the field area will be subdivided into 20 blocks of 5 × 4 cells (that is, a block is 2.5 × 2 km in size). Teams will be provided with a printed overview map of the block they are working in (Figure 1), as well as detailed high-resolution maps incorporating the QuickBird satellite data for each 500 × 500 m cell. Each team will also be provided with a handheld global positioning system (GPS) receiver (Garmin 62S; Garmin International, Inc., Olathe, KS, USA) that has been preloaded with the selected compound positions and cell boundaries. An enumeration team, comprising one field worker, a reporter and a local guide, will visit selected compounds to explain the study procedures, enumerate inhabitants, collect information on house characteristics and inform residents that the survey team will visit later that day. In situations where none of the structures within a selected compound corresponds with a residential building, the selected compound will be replaced with the nearest visible inhabited compound. The location of this replacement compound will be recorded on the satellite images, mapped using the GPS and recorded on the enumeration forms.

All compounds where at least one adult (>20 years) and one child (<15 years) are permanent residents (defined as sleeping regularly in the structure) qualify for enrolment. For compounds with fewer residents, replacements will be made, as described. If the head of the compound agrees to participate, the geographical coordinates of the main house of the compound will be recorded and compound and individual house codes will be written on the doors of all sleeping structures with a permanent marker. The names and ages of all compound members will be recorded on study forms and information on compound and house characteristics, including structure type, ITN coverage, and IRS history, will be collected using a precoded questionnaire (Programmed in Visual Basic, Visual CE v11.0) on a personal digital assistant (HP Ipaq 210, Windows Mobile 6.1). A personal study identification (ID) card will be issued to each individual, which has to be shown to the sampling team when they visit later that same day.

The field workers will carry a checklist to record the cumulative number of selected individuals for each age category. The order in which compounds are visited will be randomly selected based on a computer-generated list. After completing a compound, the enumeration team continues to the next compound until at least ten compounds have been enumerated. If the checklist indicates that age targets are not met at this point, they will continue visiting compounds according to the list until each age target is met.

#### Sampling

After enumeration, participating compounds will be visited by a sampling team consisting of two fieldworkers trained in consenting, interviewing and sampling techniques. Sampling teams will be provided with relevant maps, compound lists, enumeration forms and ID cards in advance. Compounds will be located using the names of the compound heads and by codes marked on doors at the point of enumeration; compound occupants will be asked to present their ID card for formal confirmation. Informed consenting will be conducted and the name, sex, age, residency and travel history, ITN use and sleeping times of each compound member will be recorded. The axillary temperature of each compound member will be measured by digital thermometer (Etos, Zaandam, the Netherlands). For all febrile individuals (>37.2°C axillary temperature), a rapid diagnostic test (RDT; Paracheck^®^, Orchid Biomedical Systems, Goa, India) to detect *P. falciparum*-specific histidine rich protein-2 will be performed. For all individuals surveyed, a single finger prick sample will be taken for haemoglobin (Hb) measurement using a HemoCue photometer (HemoCue 201+, Angelholm, Sweden) and three droplets of blood transferred onto a filter paper (3MM Whatman, Maidstone, UK) for serum and DNA collection. After transfer to a field laboratory, filter papers will be dried overnight and stored in plastic bags with silica gel. Once a week, samples will be transported to the Kenya Medical Research Institute (KEMRI) CDC laboratory in Kisumu and stored at −20°C until further processing. All individuals with an Hb ≤11 g/dl will be given hematinics; individuals with an Hb ≤6 g/dl will be accompanied to a nearby health centre for further care. Febrile individuals who are found to be parasitaemic by RDT will be given artemether-lumefantrine (AL, Coartem^®^, Novartis, Switzerland); women of childbearing age who are RDT positive will be assessed for pregnancy and offered a pregnancy test if deemed appropriate. Febrile children below 6 months of age and women who are suspected or found to be pregnant or are unwilling to be tested will be transported to the nearest health facility for a full assessment and treatment.

#### Malaria parasite prevalence

A combined extraction of DNA and elution of antibodies will be performed on the samples collected. Two discs with a diameter of 2.5 mm will be cut from the centre of a single filter paper bloodspot using a hole-puncher and will be eluted in deep well plates with addition of 1120 μl of a 0.5% saponin/phosphate buffered saline solution (Sigma Aldrich, Gillingham, UK). DNA will be extracted using the protocol described by Plowe *et al.*[24]; parasites will be detected by nested PCR [25,26].

#### Serological markers of malaria exposure

Total immunoglobulin G (IgG) antibodies against *P. falciparum* apical membrane antigen (AMA-1) and merozoite surface protein 1 (MSP-1_19_) will be detected by ELISA using standard methodology [27,28]. Three serological outcome measures will be used to determine spatial patterns in malaria exposure: (i) the combined antibody prevalence, that is, seropositivity for AMA-1 and MSP-1_19_ or for either of the antigens alone; (ii) the age-adjusted log_10_-transformed optical density (OD) [21,29]; (iii) the age-dependent sero-conversion rate (SCR) for combined AMA-1, MSP-1_19_ antibody prevalence [21,27].

#### Definition of hotspots

SaTScan software [30] will be used for the detection of spatial clustering in antibody prevalence (Bernouilli model) and log_10_-transformed age-adjusted OD values (normal probability model). Circular and elliptic windows [30,31] will be used to systematically scan the study area as a whole and segments of the study area using a 2 × 4 km rolling window. Hotspots will be allowed to be <1 km in radius and include <25% of the population of each window scanned. Segments of the study area will be scanned to improve the sensitivity of the scan to detect local hotspots. Local hotspots may not be detected when scanning the area as a whole, since altitude differences in the study area result in variations in average levels of transmission intensity. A hotspot will be defined as an area for which there is strong evidence (*P* < 0.05) that the observed prevalence or density of combined AMA-1 and MSP-1_19_ antimalarial antibodies is higher than expected values. Expected values are based on average values for the area as a whole and for the 2 × 4 km rolling window.

Since malaria antibodies are relatively long-lived and may indicate current as well as past malaria exposure, parasite prevalence inside and outside hotspots of malaria exposure will be determined by PCR to confirm ongoing transmission in serologically defined hotspots.

#### Selection of hotspots and evaluation areas

Since habitation in the study area is fairly evenly distributed, with every 500 × 500 m cell having six or more residential structures, clusters are unlikely to be isolated geographically. To minimize the influence of neighbouring hotspots on malaria transmission in selected intervention or control hotspots, we will select hotspots for which there are no other hotspots detected within 1 km in any direction from the hotspot boundary. The hotspot-targeted intervention will be evaluated in the area surrounding the hotspot (evaluation zones). The evaluation zone will comprise the area surrounding the hotspot up to 500 m from the hotspot boundary in each direction.

### Design of the intervention

#### Intervention clusters

Four interventions will be rolled out in the period preceding the long rainy season: larviciding, focal screening and treatment (FSAT), long-lasting insecticide-treated nets (LLIN) distribution and IRS. The details of interventions, and their timing, have been agreed upon in collaboration with the DOMC of the Kenyan Ministry of Public Health and Sanitation (MOPHS). Ten per cent of households will be visited 1 to 2 weeks after the intervention to assess any short-term side effects of the FSAT, LLINs and IRS. This sampling strategy was not based on sample size calculations but on logistical feasibility; few side effects were expected.

#### Larviciding

All permanent aquatic mosquito habitats in intervention hotspots will be mapped using handheld GPS receivers during the dry season. In the period preceding the long rainy season (April), and throughout the long rainy season (until September) all stagnant water bodies (permanent and temporary) inside these hotspots will be treated on a weekly basis with water-dispersible granule formulations of the commercial strains of *Bacillus thuringiensis var. israelensis* (Bti), VectoBac^®^, which will be provided by Valent BioSciences Corp., Libertyville, IL. Larviciding will be carried out using previously published protocols [32]; the entire hotspot area will be examined for water bodies on a weekly basis, all of which will be included in the intervention. Spot-checks for surviving *Anopheline* larvae and pupae will be made on a weekly basis.

#### Focal screen and treatment (FSAT)

All compounds in hotspots will be visited and the temperature of each individual will be determined. All individuals aged 6 months to 15 years regardless of temperature and all older individuals who are febrile (tympanic temperature ≥37.5°C) will be tested for malaria parasites using HRP-2 and pLDH based RDT (First Response^®^, Premier Medical Corporation Ltd., Kachigam, India). If one or more individuals are found to be RDT positive the entire compound will receive a curative dose of AL with the exception of pregnant women and children below 6 months of age. Because of the different times at which treatment is initiated, one treatment dose for three consecutive days will be supervised by the field worker (day 1) or community health workers (days 2 and 3). Each observed dose will be given with fatty food (>1.5 g fat) to facilitate absorption. The second daily dose will be taken without direct supervision but advice on taking the treatment with food will be given. Information on any immediate side effects of the AL will be recorded by the community health workers at each visit; all empty blister packs will be collected by community health workers after treatment has been completed to monitor adherence.

#### Long-lasting insecticide-treated nets

All compounds in hotspots will receive one LLIN per two house members according to MOPHS guidelines. LLINs (Permanet^®^ 3.0) were donated by Vestergaard Frandsen (Hanoi, Vietnam). House members will be given leaflets on proper use and maintenance of nets and study personnel will assist in hanging and demonstrate correct use of the LLINs within houses. Usage and retention of study nets will be assessed by questionnaire six weeks after distribution and any missing or badly torn nets will be replaced within two months after distribution.

#### Indoor residual spraying

Routine annual IRS with lambda cyhalothrin (ICON) will be undertaken in all structures where people are sleeping. The IRS campaigns are jointly funded by the Government of Kenya and the US President’s Malaria Initiative, and implemented by the Research Triangle Institute (RTI) with the MOPHS, DOMC and District Health Management Teams. For this study IRS will follow MOPHS protocols with more intense mobilization, repeated visits and implementation prior to the start of the malaria transmission season (March to April) in intervention hotspots.

#### Control clusters

Control clusters will receive the routine malaria control measures, which for 2012 will be the annual IRS programme as detailed and continued case management at health facilities. The IRS is scheduled to take place in April to May 2012. No LLIN distribution campaigns are planned for 2012.

### Design of the randomized evaluation

#### Sensitization and recruitment

Prior to the implementation of the interventions, meetings with district administrative and health representatives in the selected areas will be organized. Community meetings will be held with local chiefs, community elders and opinion leaders, school representatives and church leaders. All compounds in the selected intervention areas will be visited prior to the intervention; the procedures of the interventions and evaluation procedures will be explained to all compound members present. Identification cards will be distributed that will be used for identification purposes during compound visits and for identification of compound members who visit health facilities in the area.

#### Randomization

Hotspots with their surrounding evaluation areas will be randomly assigned to the intervention or control arm. This will be done by simple randomization; no stratification by parasite prevalence or altitude will be undertaken. Clusters will be ordered according to their geographical location, from northwest to southeast. Clusters will be entered in Microsoft Excel 2010 in this geographical order; the same programme will be used to generate random numbers for each of the clusters. Fifty percent of the clusters with the lowest random numbers will be assigned to the intervention arm; 50% with the highest random numbers to the control arm.

### Hypotheses and outcomes

#### Hypotheses

1. Hotspot-targeted interventions combining larviciding, LLINs, IRS and FSAT will reduce malaria transmission inside and outside hotspots of malaria transmission.

2. The community effect of hotspot-targeted interventions, defined as the impact on parasite prevalence in the evaluation zone surrounding the hotspot, is a function of distance from the hotspot boundary.

### Primary and secondary outcome measures

The primary outcome measure is parasite prevalence by PCR in the evaluation zone surrounding malaria hotspots in intervention and control clusters.

Secondary outcome measures are:

1. Parasite prevalence by PCR in the evaluation zone surrounding malaria hotspots in relation to distance to the boundary of hotspots in intervention and control clusters.

2. Indoor and outdoor Anopheles mosquito densities inside and outside hotspots of malaria transmission in intervention and control clusters.

3. The presence of Anopheles larvae in mosquito breeding sites in malaria hotspots in intervention and control clusters.

4. The number of malaria cases reporting at health facilities, coming from intervention and control clusters.

5. Reported side effects and coverage of FSAT, LLINs and IRS.

### Evaluation

#### Cross-sectional surveys

Three cross-sectional surveys will be conducted: at baseline prior to the interventions, during the peak transmission season, and at the end of the peak transmission season. For each cross-sectional survey, 25 compounds that are located inside hotspots, 25 compounds that are located <250 m from the hotspot boundary and 25 compounds that are located 250 to 500 m from the hotspot boundary will be randomly selected. This strategy is expected to give ≥100 individual observations from each of these three areas. To minimize confounding by neighbouring hotspots, an exclusion buffer will be incorporated in the selection of compounds, ensuring a minimum distance of ≥500 m from neighbouring hotspots.

Study teams will visit selected compounds and, subject to obtaining informed consent, collect information from inhabitants of all houses that belong to that compound using personal digital assistants (PDAs). For individuals older than 6 months, tympanic temperature will be measured and a finger prick blood sample (~300 μl) will be collected for assessment of haemoglobin concentration using Copack colour scales (COPACK GmbH, Oststeinbek, Germany) and for collection of nucleic acids and serum on Whatman 3MM filter paper (Maidstone, UK). Whole blood will be collected in BD K2EDTA microcontainers (BD Becton, Dickinson and Company, Oxford, UK) in selected clusters for more detailed molecular analyses. A RDT will be used to determine malaria infection for all febrile individuals. Those with a positive RDT will receive AL or will be referred to a health centre for further care.

#### Passive case detection

A passive case detection system will be introduced in government and mission health facilities to monitor individuals presenting with malaria. Facilities will be selected to cover intervention and control clusters. For this, the catchment areas of health facilities in the area have been determined. Individuals from intervention and control clusters will be asked to present a household card whenever visiting a health facility. This household card will be linked to geo-located compounds. For individuals who present without a household card, other information that allows geo-location will be collected, such as nearest school. Tympanic temperature will be measured, and an RDT used to determine parasite carriage for each individual with measured or reported fever.

#### Entomological monitoring

In a subset of the control and intervention clusters, larval and adult mosquito abundance will be monitored. Within each hotspot, a random selection of 15 water bodies along a randomly selected transect will be mapped and the presence or absence of early and late stage *Anopheline* larvae and pupae will be assessed using a 250 ml mosquito dipper. Five dips will be made in sites smaller than 5 m^2^; ten dips in sites larger than 5 m^2^. This will be carried out at two-weekly intervals. Adult collections of *Anopheline* will be carried out at the same time in 36 randomly selected houses in each cluster selected in cross-sectional surveys. Twelve of these houses will be selected within the hotspots, of which four will be sampled by pyrethrum spray catch (PSC), four for indoor light-trap collections and four for outdoor light-trap collections. Outside the hotspot 24 houses will be randomly selected of which eight will be sampled by PSC, eight for indoor and eight for outdoor light traps.

Pyrethrum spray catching will be carried out indoors according to standard WHO protocols [33]. CDC miniature light traps (Model 512; John W. Hock Company, Gainesville, FL, USA) will be used following previously published procedures to sample mosquitoes indoors [34] and outdoors [35]. The effective range of CDC light traps for outdoor mosquito sampling has been estimated as 5 m [36]. Accordingly, outdoor sampling will take place 20 m from selected houses to prevent inhabitants acting as unshielded bait. All traps will be set at 1830 hours and collected at 0630 hours. A collection bottle rotator (Model 1512, John W. Hock Company, Gainesville, FL, US) will be fitted to eight randomly selected light traps set indoors and outdoors within and outside the hotspot; this allows collection cups to rotate every two hours to estimate vector abundance at intervals throughout the night. Vector abundance, parity rates and the proportion of *Anopheline* females unfed, fed, gravid, and infected will be determined for each species [37] and compared between the two study arms.

### Statistical considerations

#### Sample size

**Figure 2:**
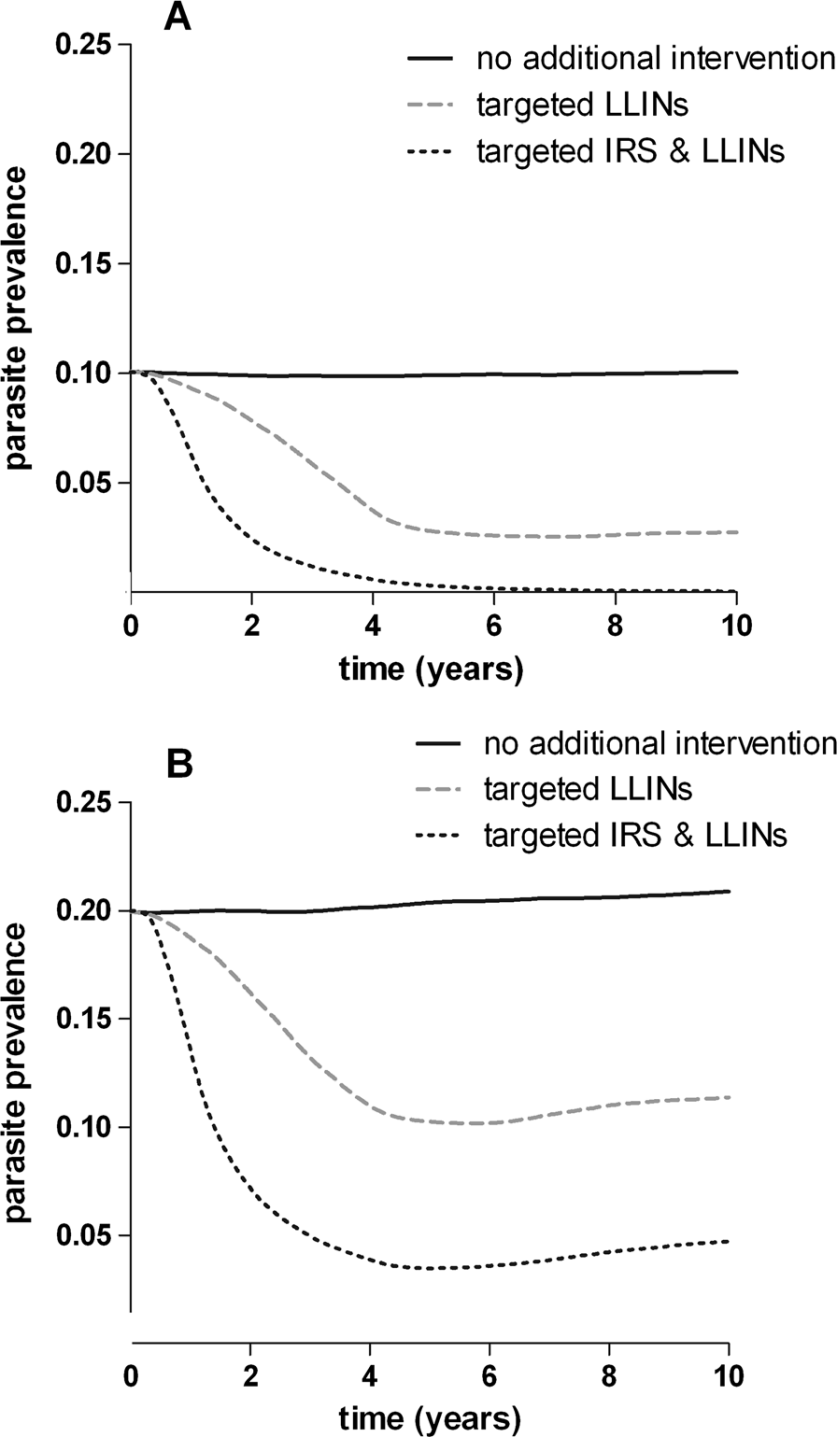
**Simulation of intervention outcome.** The figure presents a simulation of hotspot-targeted interventions in areas with a baseline parasite prevalence of 10% or 20%. ITN coverage is assumed to be 41% across all age groups (83% in under-fives). Plotted is smoothed parasite prevalence in the total population as a function of time in years since the start of the intervention. No interventions (solid black line), hotspot-targeted increase in LLIN coverage to reach 90% effective coverage in hotspots (dashed grey line) and hotspot-targeted increase in LLIN coverage to reach 90% effective coverage in hotspots in combination with targeted IRS reaching 90% of households in hotspots (dashed black line).

All available malaria simulation models indicate that malaria transmission in the area surrounding intervention hotspots will decrease considerably because malaria transmission is effectively interrupted in those compounds that seed transmission to a larger geographical area [2,16,38]. However, there are no published studies that quantify the impact of hotspot-targeted interventions. We estimated the predicted impact of targeted interventions in our study area using one of the leading individual-based simulation models [38], using human, entomological and parasitological characteristics collected at our sites in Kenya. We modelled three scenarios in situations with a pre-intervention parasite prevalence in the human population of 10 to 20%: (i) no additional interventions; (ii) targeted distribution of LLINs, reaching 90% of the population in hotspots and (iii) targeted LLINs and targeted effective IRS reaching 90% of the population in hotspots (Figure 2). The impact of larviciding is currently insufficiently described to be included in the model [38].

Our simulations show that targeted interventions can interrupt transmission completely, both inside and outside hotspots of malaria transmission, reducing overall parasite prevalence to <5%, in a manner that appears sustainable in the following years (see Figure 2). These predictions have to be interpreted with caution, since (i) the simulation model has not been prospectively tested; (ii) there is no published evidence that quantifies the impact of hotspot-targeted interventions on transmission intensity in the wider community; and (iii) the intensity of transmission will be highly variable between hotspots in our study area. There is insufficient evidence on which to base power calculations for a cluster-randomized trial; however, these simulations can give an indication of the size of the effect of the planned interventions. The primary outcome measure is parasite prevalence in the evaluation zone. A previous study on the community benefits of ITNs in Asembo, Western Kenya, indicated that an indirect beneficial effect on malaria transmission is most pronounced within 500 m of the intervention area [39]. We used this finding to define our evaluation zone surrounding the hotspots. Assuming a sample of 200 randomly selected individuals in the evaluation zone of each cluster, a coefficient of variation of true proportions between clusters within each group (*k*) of 0.4 and mean parasite prevalence of 15% and ≤5% in the control and intervention clusters respectively, would require five clusters per study arm for 80% power and 5% significance level [40]. This power calculation is based on a comparison between arms and the assumption that parasite prevalence will remain unaltered in the control arm.

To estimate the impact of the interventions on the hotspots themselves a sample size of 100 individuals in each of five clusters (hotspots) per study arm, will be required to detect a similar difference between intervention and control clusters (≤5% versus 15% mean prevalence) , assuming *k* = 0.5, 80% power and 5% significance.

The entomologic sampling approach was based on previous data from PSC studies carried out by the project where the mean number of female *Anophelines* caught per house was 0.36. To detect an 80% reduction in mean number of female *Anophelines* caught by PSC in intervention houses compared with control houses at 80% power and a significance level of 5%, 213 houses would have to be sampled in each arm. Data from light-trap studies carried out to date have shown a mean of 1.12 female *Anophelines* caught indoors per trap per night and so to detect an 80% reduction in mean number of female *Anophelines* compared with control houses at 80% power and a 5% significance level, 81 traps would have to be set in each arm per month.

### Data analysis

Statistical analyses will be performed in Stata version 12 (StataCorp LP, College Station, TX, USA). The primary analysis will be based on intention to treat whereby all evaluation areas are included in the analysis, regardless of the level of coverage. The main outcome measure, parasite prevalence, will be analyzed as binary variable. For the primary study outcome, we will compare parasite prevalence in the evaluation zones of intervention and control clusters using multilevel mixed-effects logistic regression to account for clustering per compound and random effects to account for differences between study clusters [41]. For secondary study outcomes, we will relate parasite prevalence to distance to the hotspot boundary in meters and in bins of 100 m; this analysis will be done for each of the clusters separately by generalized estimating equations (GEE), adjusting for correlations between observations from the same compound. Indoor and outdoor *Anopheles* densities will be compared between study arms using GEE models and Poisson or negative binomial distributions [42]. The proportion of productive breeding sites will be compared between intervention and control hotspots by GEE models, adjusting for correlations between observations from the same clusters.

### Ethics considerations

#### Ethics approval

The study proposal received ethics approval from the Scientific Steering Committee (SSC), the ethical review committee (ERC) of the KEMRI Nairobi (proposal numbers SSC 2163, 2181 and 1589), the London School of Hygiene & Tropical Medicine ethics committee (#6111), and from Centers for Disease Control and Prevention (with exempt status).

### Informed consent

Indoor residual spraying is to be conducted as part of the routine district-wide malaria control programme. Consent will be obtained orally at the compound by community health workers and spray operators recruited by MOPHS, as is consistent with their operating procedures. Ahead of targeted distribution of LLINs, informed, written consent will be sought at the house level from the head of the household or representative in the presence of an independent witness. Larviciding will be done after consulting with and receiving approval from the DOMC, the Kenyan Pest Control Product Board (PCPB), the district administrative, fisheries and health teams and after community meetings. Oral consent will be sought from owners of or persons responsible for any privately owned permanent breeding sites in the intervention areas (for example, fish ponds). Since most mosquito breeding sites are not restricted to particular households, consent at household level is not practical and approval from the community, DOMC and PCPB is considered adequate.

Before FSAT and cross-sectional surveys, informed written consent will be sought from all individuals and, if appropriate, their parents or guardians. If the signatory is not literate, a thumbprint will be obtained and confirmed by an independent witness. Assent forms will be signed by children between the ages of 13 and 17 years and by their parents or guardians. Each assent form will be accompanied by a consent form signed by the parent or guardian. All consent and assent forms will be countersigned by the staff member obtaining consent and a copy will be left at the household.

### Trial oversight

Ethical and safety aspects of the study are overseen by an independent monitor. No data safety and monitoring board (DSMB) will be installed. Indoor residual spraying and LLINs form part of routine malaria control in Kenya and will be undertaken in collaboration with the DOMC and the district public health teams. Larviciding with Bti has been undertaken previously in neighbouring districts and has previously been shown to pose no health risk [43]. The proposed form of FSAT, where household members of parasite carriers are treated regardless of their parasite status by microscopy, is not part of the current malaria strategy of the Kenyan DOMC, although screening and treatment of asymptomatic parasite carriers is recommended [44]. Our FSAT approach is based on the assumption of a high proportion of submicroscopic infections among asymptomatic individuals [45], especially among household members of individuals with patent parasitaemia [46]. The drug used throughout the study, AL, is the first line antimalarial treatment in most of East Africa, including Kenya.

## Discussion

Targeting interventions to hotspots of malaria transmission is frequently mentioned as a cost-effective approach for malaria control and elimination [2,4,5,47], although direct evidence for a community effect of hotspot-targeted interventions is currently unavailable. The present study aims to determine this effect in a cluster-randomized intervention trial.

Valuable information on how to quantify community effects of malaria control interventions comes from trials with ITNs [48]. Mortality rates [49], incidence of severe malaria [50], incidence of uncomplicated malaria [39,50], anaemia [39] and high-density parasitaemia [39] have been shown to be reduced in compounds without ITNs that were in close proximity of compounds with ITNs. Hawley and colleagues found that individuals living in control villages within 300 m of ITN villages in Kenya experienced a level of protection similar to that experienced by individuals living in ITN villages and that this was plausibly due to area-wide effects on vector densities and sporozoite-positive mosquitoes [39]. Despite similarities, hotspot-targeted interventions may differ considerably from untargeted ITN campaigns in their community impact. Mathematical simulation models suggest that the impact of hotspot-targeted interventions may be much larger than that of community-wide ITN distributions and may lead to local malaria elimination [4]. In line with this, our trial is powered to detect large effects on malaria transmission. However, two of the major assumptions underlying the optimistic model outcomes are incompletely understood. Firstly, the stability of hotspots is central to ensure sustainable community effects. Hotspots of (asymptomatic) parasite carriage are generally assumed to be stable [4,10]. However, a report that wind direction in relation to breeding site location may be a key element in determining the location of hotspots [12], suggests that local environmental factors may also influence the spatial stability of hotspots. We believe that our approach to define hotspots serologically may be less susceptible to (short-term) variations in wind direction or other ecological factors, since it effectively bases hotspot-detection on immunological markers of cumulative malaria exposure [27]. Secondly, a community effect of hotspot-targeted interventions strongly depends on mosquito mixing patterns. Mosquito mixing patterns are unlikely to be homogeneous. Reported site-fidelity where mosquitoes are likely to return to the same compounds [51,52] remains to be confirmed but could considerably reduce the community effect of hotspot-targeted interventions. The most informative measure of mixing patterns may be an approach where parasite populations are tracked in human populations, inside and outside hotspots of malaria transmission.

Research on the impact of community interventions where ‘herd coverage’ is required to ensure effectiveness raises a number of practical issues. Similar to mass drug administration campaigns, high community coverage [53,54] is required in our study to reduce *R*_0_ to values below 1. Our intervention is further challenged by a dependence on community participation in control measures that are only rolled out in a selected proportion of this community. Gaining community trust is essential to the study’s success and we expect good participation rates after our lengthy sensitization process and strong involvement of community leaders and local workers in all aspects of the study preparation, intervention and evaluation.

Even with excellent participation rates, the nature of our intervention will remain susceptible to contamination from neighbouring hotspots. An ideal study setting would comprise a large number of geographically isolated clusters, each being an independent focus of malaria transmission, with clearly defined hotspots within these clusters [4]. Our real-life setting falls short of this ideal scenario. The continuous inhabitation in the area makes it unlikely that clusters are geographically isolated. We aim to minimize contamination from non-targeted malaria hotspots by incorporating an exclusion zone in our selection of eligible hotspots and in the selection of compounds in the evaluation phase. We nevertheless expect that there will be residual contamination that will be reflected in a spatial component in the effect of hotspot-targeted interventions: we expect the level of contamination to be highest in areas furthest away from the targeted hotspot and nearest to untargeted hotspots. Similarly, the effect of the intervention within the targeted hotspots may be largest in those compounds that are most remote from the nearest untargeted compound. Mathematical simulation models that incorporate heterogeneous malaria exposure [16,23,38] are expected to be valuable as an integral part of the evaluation of our intervention to assess the plausibility that a change in transmission intensity can be attributed to the intervention.

The current study is not designed to determine the effect of individual interventions. While simulations suggest that targeted interventions with LLINs and IRS will be sufficient to eliminate malaria locally [4], we chose a relatively comprehensive package of malaria control measures incorporating a wide variety of available interventions, targeting both the mosquito vector and the malaria parasite in humans. If findings from the current study prove promising, a next step will be to determine the optimum package of tools for hotspot-targeted interventions across a range of settings. This package will differ between different settings. Larviciding, for example, will be most beneficial in settings where breeding sites are discrete and well-defined [55-57] while the effects of IRS and ITNs will be affected by insecticide resistance, amongst other factors [48]. Importantly, follow-up studies should determine the cost-effectiveness of the hotspot approach to assess whether savings in the number of compounds that need to be targeted for conventional vector control in the absence of hotspot treatment outweigh the costs for hotspot-detection and coordination of hotspot interventions.

## Trial status

The trial was actively recruiting participants at the time that the protocol was submitted for publication.

## Abbreviations

ACT: artemisinin combination therapy; AL: artemether-lumefantrine; AMA-1: apical membrane antigen; Bti: *Bacillus thuringiensis israelensis*; CDC: Centers for Disease Control; DOMC: (Kenyan) Division of Malaria Control; DSMB: Data Safety and Monitoring Board; EIR: entomologic inoculation rate; ELISA: enzyme-linked immunosorbent assay; ERC: ethical review committee; FSAT: focal screen and treat; GEE: generalized estimating equations; GPS: global positioning system; Hb: haemoglobin; HRP-2: histidine rich protein-2; ICON: lambda cyhalothrin (ICON 10 CS); ID: identification; IgG: immunoglobulin G; IRS: indoor residual spraying; ITN: insecticide-treated net; KEMRI: Kenya Medical Research Institute; LLIN: long-lasting insecticide-treated net; MOPHS: Ministry of Public Health and Sanitation; MSP-1: merozoite surface protein 1; OD: optical density; PCPB: Kenyan Pest Control Product Board; PCR: polymerase chain reaction; PDA: personal digital assistant; pLDH: plasmodium lactate dehydrogenase; PSC: pyrethrum spray catch; *R*_0_: basic reproduction number; RDT: rapid diagnostic test; RTI: Research Triangle Institute; SCR: sero-conversion rate; SSC: Scientific Steering Committee.

## Competing interests

The authors declare that they have no competing interests.

## Authors’ contributions

Trial design: TB, JS, IK, RS, CD, JC; design of intervention packages JS, AB, GS, JV, NB, KL, MD, JC; design of analytical plan: TB, JTG, IK, KL, MD, RS, CD, JC; preparation and conduct of surveys: JS, AB, GS; contribution of reagents EJR, contributed to manuscript preparation TB, JS, AB, GS, JTG, IK, EJR, JV, NB, MD, RS, CD, JC. All authors read and approved the final manuscript.
